# Novel complex translocation involving 5 different chromosomes in a chronic myeloid leukemia with Philadelphia chromosome: a case report

**DOI:** 10.1186/1755-8166-2-21

**Published:** 2009-11-09

**Authors:** Walid Al Achkar, Abdulsamad Wafa, Hasmik Mkrtchyan, Faten Moassass, Thomas Liehr

**Affiliations:** 1 Molecular Biology and Biotechnology Department, Human Genetics Div., Atomic Energy Commission of Syria, P.O. Box 6091, Damascus, Syria; 2 Jena University Hospital, Institute of Human Genetics and Anthropology, Kollegiengasse 10, D-07743 Jena, Germany

## Abstract

**Background:**

The well-known typical fusion gene BCR/ABL can be observed in connection with a complex translocation event in only 2-10% of cases with chronic myeloid leukemia (CML). As currently most CML cases are treated with Imatinib, variant rearrangements have in general no specific prognostic significance, though the emergence of therapy resistance remains to be studied.

**Results:**

Here we report an exceptional CML case with complex chromosomal aberrations not observed before, involving a 5 chromosome translocation implying chromosomal regions such as 1q42, 4p14 and 5q31 besides 9q34 and 22q11.2.

**Conclusion:**

The reported rearrangement developed most probably in one initial step and had no influence on a good response during Imatinib treatment.

## Introduction

Chronic myeloid leukemia (CML), a clonal myeloproliferative disease is known to develop from a pluripotent bone-marrow stem cell following the typical BCR and ABL somatic gene rearrangement. In 90-95% of cases with CML, the BCR/ABL fusion gene is the result of reciprocal translocation between chromosomes 9 and 22 and is cytogenetically observable as a small derivative chromosome 22 which is known as Philadelphia (Ph1) chromosome [1,2].

In a Ph-positive CML expression of the BCR/ABL chimeric protein p210 with an increased tyrosine kinase activity is essential for multiple signaling pathways to confer the leukemia phenotype [3]. Imatinib mesylate (Glivec, formerly STI571) was designed specifically to inhibit the tyrosine kinase activity of the bcr/abl protein and other tyrosine kinases such as c-abl, c-kit and platelet-derived growth factor receptor. By binding to an active site of the tyrosine kinase, Imatinib mesylate switches off downstream signaling, cells stop proliferating and apoptosis ensues [4]. Many studies have shown a high efficiency of Imatinib therapy to achieve a complete or major cytogenetic response, i.e. 0-34% Ph-positive cells. This positive effect may be achieved in cases with simple t(9;22), and complex translocations resulting in a BCR/ABL fusion gene, as well as in cases with cytogenetic clonal evolution [5,6].

Complex chromosomal rearrangements involving one or more additional chromosomes were described in >600 cases with CML [7]. By conventional cytogenetic analysis, two variant subgroups have traditionally been recognized: complex, t(9;22;V), where V represents a third translocation partner chromosome and simple, t(9;V) or t(22;V) [8]. Only in a few cases is a chromosomal fragment from the third chromosome translocated to the der(22)t(9;22), producing a 'masked Ph' [9]. In most Ph-variant cases the segment 22q11->qter is moved to a third chromosome, while a part of the third chromosome is located on 9q34. Deletions on the derivative chromosome 9 were found to occur with a much higher frequency in patients with variant Ph translocations (45%) than in those with classic Ph (17%) [10].

Herein we report on a CML cases with new complex aberrations with five chromosomal breakpoints, nonetheless successfully treatable by Imatinib.

## Case report

The 45 year old female patient presented initially with a whole blood cell count (WBC) of 43.500/l, splenomegaly and severe loss of weight. Chromosome analysis using banding cytogenetics only revealed a karyotype in concordance with the clinical diagnosis of a CML in chronic phase. She was treated with Hydroxyurea (1000 mg daily dose).

Four years and four months later her WBC was 17.61 × 10^9^/l, i.e. 71.6% neutrophils, 18.9% lymphocytes, 3.1% eosinophiles, 5.1% monocytes, 0.2% basophiles. Platelets count was 650 × 10^9^/l and hemoglobin 12.1 g/dl. She was treated with Imatinib (400 mg daily dose) for overall 10 months. Then her WBC was 27.70 × 10^9^/liter, i.e. 70.2% neutrophils, 16.5% lymphocytes, 5.7% eosinophiles, 6.6% monocytes and 0.1% basophiles. Platelets count was 793 × 10^9^/l and hemoglobin 12.7 g/dl. Serum lactate dehydrogenase (LDH) was 515 U/l (normal up to 480 U/l), and serum alkaline phosphates was 120 U/l (normal: up to 90 U/l).

**Figure 1:**
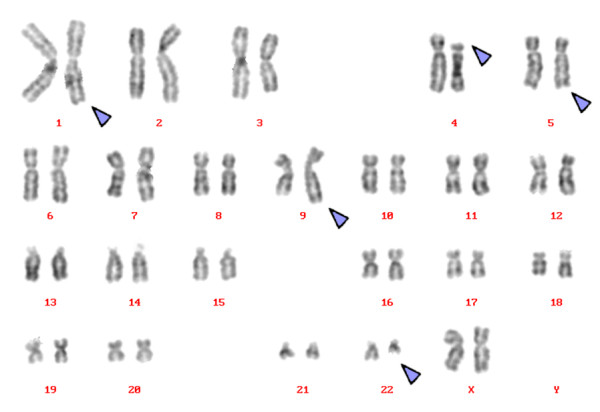
**GTG-banding revealed a complex karyotype involving three further chromosomes besides chromosomes 9 and 22**. All derivative chromosomes are marker by arrowheads.

**Figure 2:**
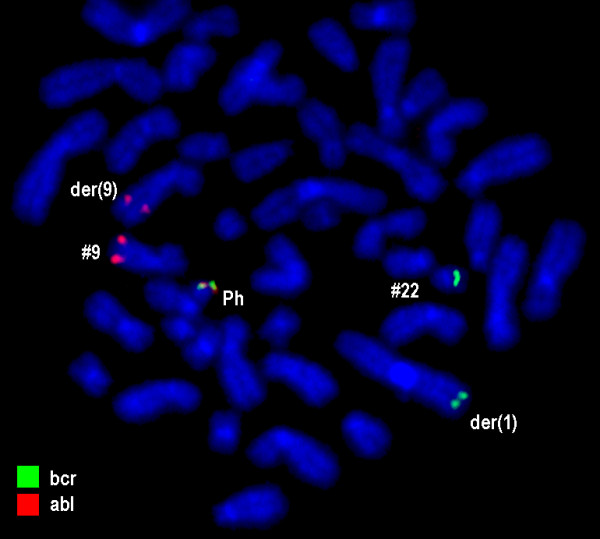
**Fluorescence in situ hybridization (FISH) using probes for BCR (green) and ABL (red) confirmed an involvement of chromosome 1 in the rearrangement present in this case**. Abbreviations: # = chromosome; der = derivative chromosome; Ph = Philadelphia-chromosome.

**Figure 3:**
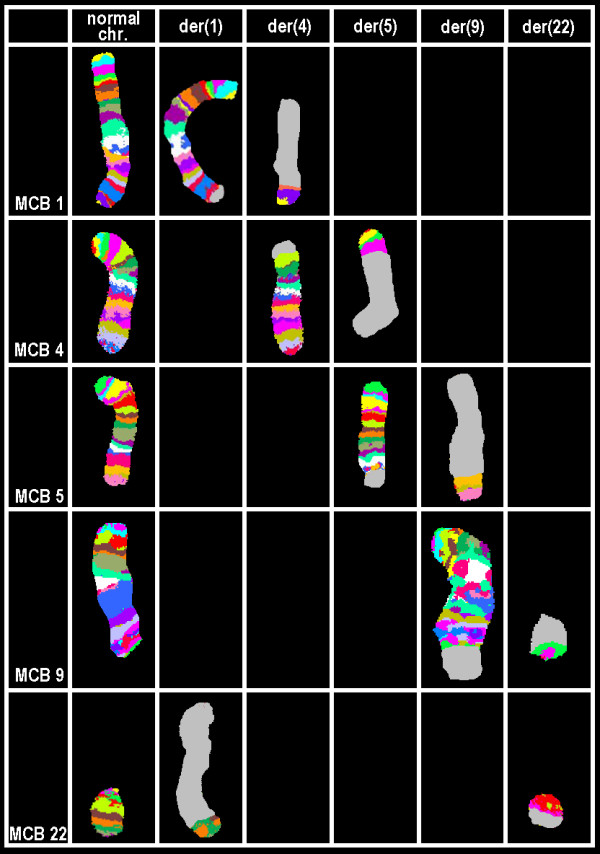
**Array-proven multicolor banding (aMCB) was applied to determine the involved in this complex rearrangement**. In each lane the results of aMCB analysis using probe-sets for chromosomes 1, 4, 5, 9 and 22 are shown. The normal chromosomes are shown in the first column, the derivative of all five chromosomes in the following ones. In the light gray by aMCB-probes unstained regions on the derivative chromosomes are depicted.

Karyotyping was done again at 3 and 10 months after initiation of Imatinib-treatment, respectively, showing additional karyotypic changes. A complex karyotype 46, XX, t(1;4;5;9;22) was determined in GTG-banding (Fig. 1) and further studied by molecular cytogenetics (Fig. 2 and Fig. 3). Dual-color-FISH using a commercially available probe specific for BCR and ABL revealed that the typical Philadelphia-chromosome with BCR/ABL-translocation was present. However, parts of chromosome 22 were present on a der(1) (Fig. 2). Thus, array-proven high-resolution multicolor banding (aMCB, Fig. 3), using probes for the corresponding chromosomes involved according to GTG-banding, was done [11]. The following result was obtained: 46, XX, t,(1;4;5;9;22)(q42;p14;q31;q34;q11.2). Thus, it is most likely that the complexity of the karyotype was missed initially due to low chromosomal resolution.

## Discussion

According to the literature, in 2-10% CML cases the fusion gene BCR/ABL is a result of a complex translocation. At present it appears that variant translocations can affect any chromosome. However, it has been suggested that distribution of the break-points is non-random with the chromosomal bands most susceptible to breakage being: 1p36, 3p21, 5q31, 6p21, 9q22, 10q22, 11q13, 12p13, 17p13, 17q21, 17q25, 19q13, 21q22, 22q12 and 22q13 [7]. In our study we found one out of the three breakpoints to be member of this list, i.e. 5q31. However, the fusion gene remained on chromosome 22.

Two possible mechanisms for variant translocation formation were suggested. The first is a single-event rearrangement via the simultaneous breakage of several chromosomes followed by mismatched joining [12]. Nacheva et al proposed a classical Ph translocation followed by a further translocation event between chromosomes 9 and 22 plus a third chromosome [13]. The mechanism of the formation of a variant Ph translocation may have prognostic importance in that a two-event mechanism represents clonal evolution, whereas a variant translocation occurring via a single genomic rearrangement may confer a similar prognosis to the classical Ph translocation; i.e. clonal evolution has a worse prognosis [14]. According to that, we suggest that the reported patient developed the rearrangement in one initial step and thus, also had such a good response to treatment.

## Materials and methods

Banding cytogenetics using GTG-banding was done according to standard procedures [15]; 20 metaphases derived from unstimulated bone marrow of the patient were analyzed, each.

Fluorescence in situ hybridization (FISH) using commercially available probes for BCR and ABL (Abbott/Vysis) were applied according to manufacturers instructions. High resolution array-proven multicolor-banding (aMCB) based on microdissection derived region-specific libraries for chromosome 1, 4, 5, 9 and 22 was done as described before; method and MCB probe sets are specified [11,16]. 20 metaphase spreads were analyzed, each using a fluorescence microscope (AxioImager.Z1 mot, Zeiss) equipped with appropriate filter sets to discriminate between a maximum of five fluorochromes and the counterstain DAPI (Diaminophenylindol). Image capturing and processing were carried out using an Isis mFISH imaging system (MetaSystems, Altlussheim, Germany) for the evaluation MCB.

## Competing interests

The authors declare that they have no competing interests.

## Authors' contributions

AW and FM performed the cytogenetic studies in the present case and collected the data relative to this case report. WA supervised the cytogenetic analysis as Director of the MBBD. HM, AW, FM TL did the molecular cytogenetic analysis and interpretation. TL drafted the paper and all authors contributed to the finalizing of the manuscript.

## Consent

Written informed consent was obtained from the patient for publication of this case report and accompanying images. A copy of the written consent is available for review by the Editor-in-Chief of this journal.

## References

[B1] Rooney DE (2001). Human Cytogenetics Malignancy and Acquired Abnormalities.

[B2] Sessions J (2007). Chronic myeloid leukemia in 2007. Am J Health Syst Pharm.

[B3] Lugo T, Pendergast A, Müller A, Witte O (1990). Tyrosine kinase activity and transformation potency of bcr-abl oncogene products. Science.

[B4] Griffen J (2001). The biology of signal transduction The biology of signal transduction inhibition: basic science to novel therapies. Semin Oncol.

[B5] Kantarjian H, Sawyers C, Hochhaus A, Guilhot F, Schiffer C, Gambacorti-Passerini C, Niederwieser D, Resta D, Capdeville R, Zoellner U, Talpaz M, Druker B, Goldman J, O'Brien SG, Russell N, Fischer T, Ottmann O, Cony-Makhoul P, Facon T, Stone R, Miller C, Tallman M, Brown R, Schuster M, Loughran T, Gratwohl A, Mandelli F, Saglio G, Lazzarino M, Russo D, Baccarani M, Morra E (2002). Hematologic and cytogenetic responses to imatinib mesylate in chronic myelogenous leukemia. N Engl J Med.

[B6] Cortes JE, Talpaz M, Giles F, O'Brien S, Rios MB, Shan J, Garcia-Manero G, Faderl S, Thomas DA, Wierda W, Ferrajoli A, Jeha S, Kantarjian HM (2003). Prognostic significance of cytogenetic clonal evolution in patients with chronic myelogenous leukemia on imatinib mesylate therapy. Blood.

[B7] Johansson B, Fioretos T, Mitelman F (2002). Cytogenetic and molecular genetic evolution of chronic myeloid leukemia. Acta Haematol.

[B8] Huret JL (1990). Complex translocations, simple variant translocations and Ph-negative cases in chronic myelogenous leukaemia. Hum Genet.

[B9] Hagemeijer A, de Klein A, Godde-Salz E, Turc-Carel C, Smit EME, van Aghtoven AJ, Grosveld GC (1985). Translocation of c-abl to 'masked' Ph in chronic myeloid leukemia. Cancer Genet Cytogenet.

[B10] Reid A, Gribble SM, Huntly BJ, Andrews KM, Campbell L, Grace CD, Wood ME, Green AR, Nacheva EP (2001). Variant Philadelphia translocations in chronic myeloid leukaemia can mimic typical blast crisis chromosome abnormalities or classic t(9;22): a report of two cases. Br J Haematol.

[B11] Weise A, Mrasek K, Fickelscher I, Claussen U, Cheung SW, Cai WW, Liehr T, Kosyakova N (2008). Molecular definition of high-resolution multicolor banding probes: first within the human DNA sequence anchored FISH banding probe set. J Histochem Cytochem.

[B12] Fitzgerald PH, Morris CM (1991). Complex chromosomal translocations in the Philadelphia chromosome leukemias. Serial translocations or a concerted genomic rearrangement?. Cancer Genet Cytogenet.

[B13] Nacheva E, Holloway T, Brown K, Bloxham D, Green AR (1994). Philadelphia-negative chronic myeloid leukaemia: detection by FISH of BCR-ABL fusion gene localized either to chromosome 9 or chromosome 22. Br J Haematol.

[B14] Reid AG, Huntly BJP, Grace C, Green AR, Nacheva EP (2003). Survival implications of molecular heterogeneity in variant Philadelphia-positive chronic myeloid leukaemia. Br J Haematol.

[B15] Claussen U, Michel S, Mühlig P, Westermann M, Grummt UW, Kromeyer-Hauschild K, Liehr T (2002). Demystifying chromosome preparation and the implications for the concept of chromosome condensation during mitosis. Cytogenet Genome Res.

[B16] Liehr T, Heller A, Starke H, Rubtsov N, Trifonov V, Mrasek K, Weise A, Kuechler A, Claussen U (2002). Microdissection based high resolution multicolor banding for all 24 human chromosomes. Int J Mol Med.

